# Uniparental Genetic Heritage of Belarusians: Encounter of Rare Middle Eastern Matrilineages with a Central European Mitochondrial DNA Pool

**DOI:** 10.1371/journal.pone.0066499

**Published:** 2013-06-13

**Authors:** Alena Kushniarevich, Larysa Sivitskaya, Nina Danilenko, Tadeush Novogrodskii, Iosif Tsybovsky, Anna Kiseleva, Svetlana Kotova, Gyaneshwer Chaubey, Ene Metspalu, Hovhannes Sahakyan, Ardeshir Bahmanimehr, Maere Reidla, Siiri Rootsi, Jüri Parik, Tuuli Reisberg, Alessandro Achilli, Baharak Hooshiar Kashani, Francesca Gandini, Anna Olivieri, Doron M. Behar, Antonio Torroni, Oleg Davydenko, Richard Villems

**Affiliations:** 1 Estonian Biocentre, Tartu, Estonia; 2 Institute of Genetics and Cytology, National Academy of Sciences of Belarus, Minsk, Belarus; 3 Belarusian State University, Faculty of History, Department of Ethnology, Museology and History of Arts, Minsk, Belarus; 4 Centre of Forensic Expertise and Criminalistics, Minsk, Belarus; 5 Department of Evolutionary Biology, Institute of Molecular and Cell Biology, University of Tartu, Tartu, Estonia; 6 Human Genetics Group, Institute of Molecular Biology, Academy of Sciences of Armenia, Yerevan, Armenia; 7 Dipartimento di Biologia Cellulare e Ambientale, Università di Perugia, Perugia, Italy; 8 Dipartimento di Biologia e Biotecnologie “L. Spallanzani”, Università di Pavia, Pavia, Italy; 9 Molecular Medicine Laboratory, Rambam Health Care Campus, Haifa, Israel; 10 Estonian Academy of Sciences, Tallinn, Estonia; University of Cambridge, United Kingdom

## Abstract

Ethnic Belarusians make up more than 80% of the nine and half million people inhabiting the Republic of Belarus. Belarusians together with Ukrainians and Russians represent the East Slavic linguistic group, largest both in numbers and territory, inhabiting East Europe alongside Baltic-, Finno-Permic- and Turkic-speaking people. Till date, only a limited number of low resolution genetic studies have been performed on this population. Therefore, with the phylogeographic analysis of 565 Y-chromosomes and 267 mitochondrial DNAs from six well covered geographic sub-regions of Belarus we strove to complement the existing genetic profile of eastern Europeans. Our results reveal that around 80% of the paternal Belarusian gene pool is composed of R1a, I2a and N1c Y-chromosome haplogroups – a profile which is very similar to the two other eastern European populations – Ukrainians and Russians. The maternal Belarusian gene pool encompasses a full range of West Eurasian haplogroups and agrees well with the genetic structure of central-east European populations. Our data attest that latitudinal gradients characterize the variation of the uniparentally transmitted gene pools of modern Belarusians. In particular, the Y-chromosome reflects movements of people in central-east Europe, starting probably as early as the beginning of the Holocene. Furthermore, the matrilineal legacy of Belarusians retains two rare mitochondrial DNA haplogroups, N1a3 and N3, whose phylogeographies were explored in detail after *de novo* sequencing of 20 and 13 complete mitogenomes, respectively, from all over Eurasia. Our phylogeographic analyses reveal that two mitochondrial DNA lineages, N3 and N1a3, both of Middle Eastern origin, might mark distinct events of matrilineal gene flow to Europe: during the mid-Holocene period and around the Pleistocene-Holocene transition, respectively.

## Introduction

Contemporary Belarus occupies the central-western fringe of the East European plain. Its current landscape is due to features acquired from glacier activities, which finally retreated around 12 thousand years ago (kya) [1]. Starting from this period, the Belarusian territory is thought to have been completely and continuously populated by Anatomically Modern Humans (AMH). However, the prehistoric period *per se* lasted longer since the earliest evidence of AMH activities in the present territory of Belarus are dated to the Middle Upper Paleolithic period (20–25 kya) [2], [3].

Human genome variation has been successfully used to reconstruct the lengthy prehistory of human populations and two genetic loci, in particular, the non-recombining portion of the Y-chromosome (NRY) and mitochondrial DNA (mtDNA), have proven to be very informative [4]–[7]. The uniparental gene pools have been explored at different levels among eastern Europeans. Previous studies have shown that the maternal gene pool in East Europe is composed of typical West Eurasian haplogroups and is characterized by very similar compositions among populations [8]–[14]. Studies on Y-chromosome indicate the composite regional background of paternal gene pools (e.g. R1a, I lineages) and the substantial substructure of eastern European populations [15]–[29].

There are only a few studies performed at a low level of molecular resolution and based on restricted sampling, which target common NRY and mtDNA variation in Belarusians [30], [31] and infer the intra-population structure using Y-chromosome Short Tandem Repeats (Y-STRs) [24], [32]. Hence, comprehensive profiling of the gene pool of Belarusians within central-east Europeans along with possible sources of prevalent and rare uniparental lineages has remained unexplored.

In this study, we aim to unravel the genetic structure of Belarusians using high resolution analysis of 565 Y-chromosomes and 267 mtDNAs representing six geographic sub-regions of Belarus and to evaluate the temporal and geographic origin of their most common and rare lineages. Furthermore, we studied in detail the phylogeny and phylogeography of two common Belarusian NRY lineages (N1c(Tat) and I2a(P37)) and, at the level of complete mitogenomes, we investigated two deeply rooted mtDNA haplogroups (N1a3 and N3), which are generally rare but were observed in Belarusians and therefore are potentially informative from the phylogeographic perspective.

## Results and Discussion

### Maternal gene pool of Belarusians

**Figure 1:**
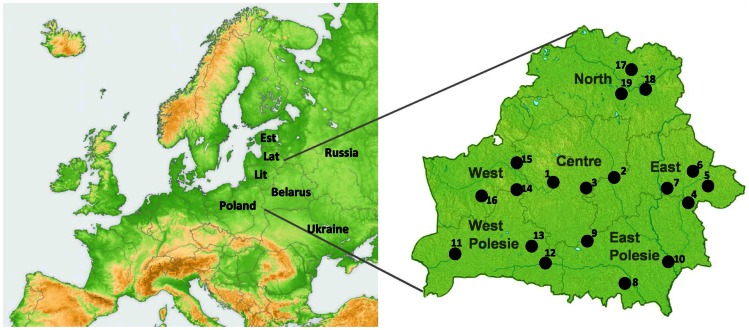
Geographic position of Belarus within Europe. Map of Belarus demonstrating the six geographic sub-regions studied is shown on the right. Numbers 1–19 correspond to the location of sampling points (Table S1). Lit – Lithuania, Lat – Latvia, Est – Estonia.

**Figure 2:**
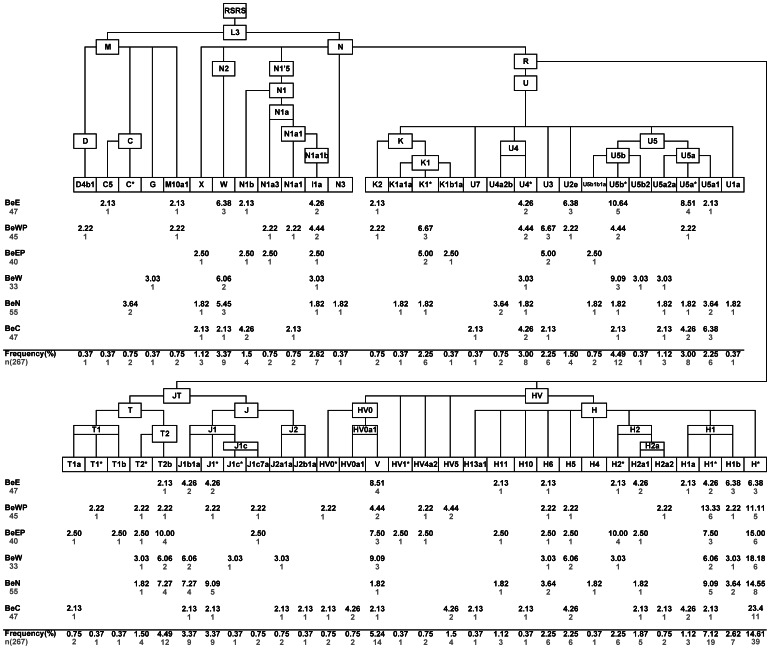
Phylogeny of mtDNA haplogroups and their relative frequencies in Belarusians. The tree is rooted relative to the RSRS according to [51]. Belarusian sub-populations are designated as BeE – East, BeWP – West Polesie, BeEP – East Polesie, BeN – North, BeC – Centre, BeW – West. Sample sizes and absolute frequencies are also given.

A total of 267 individuals from six geographic regions of Belarus (Figure 1, Table S1) were included in the study of mtDNA diversity. Fifty-eight sub-haplogroups, as well as paraphyletic groups within them, were identified, all descending from the two basal Eurasian mtDNA haplogroups M and N(R) (Figure 2, Table S2).

Majority of the detected haplogroups are those diversified primarily within Europe and those characterizing the central-east European mtDNA pool. Among all H sub-branches identified, H1b and H2a are well represented (Figure 2), which is in agreement with previously reported studies of eastern European populations [33], [34]. Belarusian haplogroup V is characterized by three first hypervariable segment (HVS-I) haplotypes (151–153 in Table S2), which are more prevalent in East Europe than in West Europe [11], [26], [35], [36]. Similarly, U5a, another frequent haplogroup, is shown to be more typical for eastern Europeans compared to central and south-eastern ones, whereas haplogroup U5b reflects the input from south-west and Central Europe [37]. Certain Belarusian U4 sub-haplogroups, e.g. U4a2, are shown to be well represented among other Slavic-speaking populations of central-east Europe and to have expanded during the last 5–8 ky [38]. Haplogroups J and T include those haplotypes that have been shown to be European as well as Near-Eastern specific (Figure 2) [39]. It was suggested that particular branches within J and T were in Europe already during the Late Glacial period, whereas only a few sub-lineages showed signs of Neolithic input from the Near East [39].

Less frequent in Belarusians are mtDNA haplogroups stemming directly from the N-node (except the R-branch), suggesting a genetic contribution from Near/Middle East region. The N1a1 HVS-I motif (haplotype 95 in Table S2) detected in Belarusians has been shown to be common among first farmers in Central Europe, but rare in the preceding Paleolithic/Mesolithic populations and in modern Europeans [40], [41]. Haplogroup I1a is another lineage most likely associated with the Neolithic, whereas haplogroup W seems to have expanded within Europe earlier [42]. N1a3 and N3 mtDNAs are also likely evidences of Middle Eastern genetic traces in Europeans.

The smallest component of the Belarusian maternal gene pool comprises of four East Asian specific M-rooted haplogroups (Figure 2). It has been demonstrated that such a minor share of East Eurasian mtDNAs is steady among western and central-east Europeans irrespective of their linguistic affiliation, while it increases notably eastward within East Europe reaching up to one-third among particular Volga-Uralic populations [13], [43].

To visualize the intra-population structure of Belarusians we used principle component (PC) analysis based on haplogroup frequencies. West and East Polesie (southern Belarus) are scattered in the PC plots and shifted from other sub-regions due to lower frequencies of haplogroups J and U5 and higher frequencies of T, H2 and K (PC plots PC1*vs*PC2 and PC1*vs*PC3 in Figure S1). However, this could be, partly, due to events of genetic drift within these relatively small regions. MtDNA haplogroups in general demonstrate notable frequency patterns only in a wider geographic context, e.g. within West Eurasia [7], [33], [39], whereas within a smaller region, such as the East European plain, these patterns are not seen for all lineages (Table S3). We examined the genetic variance between two major geographical subdivisions in Belarusians, in particular, southern *vs* the remaining four sub-populations and western *vs* the remaining sub-groups (see the Methods section for grouping details) using analysis of molecular variance (AMOVA). Our results suggest that the genetic differentiation between southern and the rest of the sub-groups was marginally significant (Table S4) although the inter-group variation was low (0.32%). In addition, pairwise Fst values tested between six Belarusian sub-populations indicate very low inter-population genetic differentiation (Table S5). Altogether, our mtDNA analyses suggest that there is no strong evidence of substructure within the Belarusian maternal gene pool.

**Figure 3:**
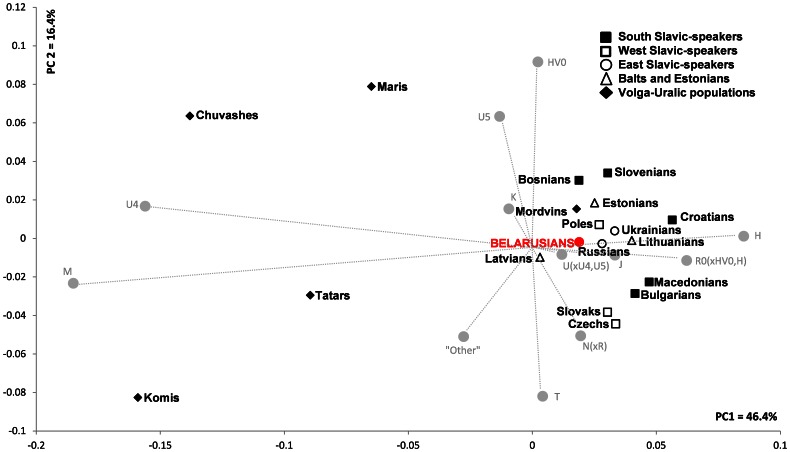
PC analysis based on mtDNA haplogroup frequencies among eastern Europeans and Balkan populations. The contribution of each haplogroup to the first and the second PCs is shown in gray. The group “Other” includes “Other” from published data merged with uncommon haplogroups L1b, L2a and L3f. Frequencies of mtDNA haplogroups and references are listed in Table S3.

Frequencies of Belarusian mtDNA haplogroups do not differ considerably from other eastern European and Balkan populations, at least when major clades such as H1, H2, V, U5a and U5b, K, T and J are considered (Table S3). However, populations from the easternmost fringe of the eastern European region, the Volga-Uralic, have a decreased share of overall H mtDNAs and a noticeably increased frequency of haplogroup U4 as well as M-lineages compared to Belarusians (Table S3). Our first PC analysis based on mtDNA haplogroup frequencies revealed that Belarusians along with majority of other eastern European and Balkan populations formed a single cluster while the members of the Volga-Uralic region except Mordvins are separated from rest of the studied populations with Udmurts and Bashkirs being the most remote on the plot, as expected due to their distinctive genetic compositions (Figure S2). Hence, to increase the resolution within the former group, we performed the second PC analysis excluding Bashkirs and Udmurts (Figure 3). In the resulting PC plot the populations clustered more or less according to their geographic affinities with Belarusians grouping together with their immediate neighbors - Russians, Ukrainians, Poles and Lithuanians, and being somewhat separated from Czechs and Slovaks as well as representatives of the Balkan region (Figure 3).

### Revision of the mtDNA N1a3 phylogeny

Two Belarusian mtDNAs, characterized by an unusual HVS-I motif (A16240c, A16265G from the N-root), were classified as members of N1a3 based on the A16265G transition (Table S2). MtDNAs bearing A16265G along with C16201T relative to the N-node were assigned to haplogroup N1a3 (ex-N1c) [44] for the first time in [6] and since then this was considered as the diagnostic motif for N1a3.

At the level of mtDNA control-region variation, the geographic distribution of N1a3 is well described to date: it is primarily confined to the Middle East, with the highest frequencies but a rather low diversity, as far as HVS-I is concerned, in populations of the Arabian Peninsula [42], [45] and in the Marsh Arabs of Iraq [46]. It is also present in the Caucasus region, most frequently among Armenians, but also among Georgians, as well as in Adygei and Dagestan people of the North Caucasus (our unpublished data). N1a3 is found throughout the north-east of the Mediterranean basin (Sicily, Rhodes, Crete, Cyprus, among Lebanese and Palestinians) and in the Turkish Kurds ([47] and our unpublished data). It is extremely rare in central-east Europe: single mtDNAs have been found among Romanians, Poles, Belarusians and among populations of the Volga-Ural region, Tatars and Mordvins ([12], [13], [48], [49] and our unpublished data). We note that unusually high incidence of the N1a3 among Mordvins, living in the East European plain of Russia, is meanwhile characterized by low HVS-I diversity, indicative of a possible founder event in recent times.

**Figure 4:**
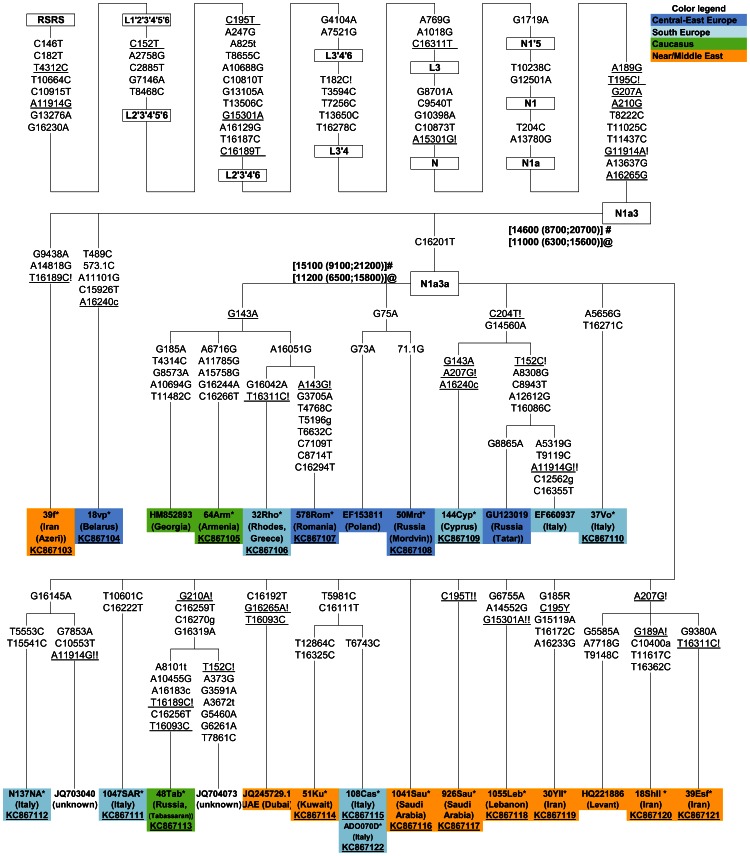
Maximum parsimony tree of mtDNA haplogroup N1a3. The tree includes 20 novel complete sequences (marked with an asterisk and underlined accession numbers) and eight previously published [42], [50(and references therein)], [51]. Mutations relative to the RSRS [51] are indicated on the branches; transversions are specified with a lower case letter; Y and R stand for heteroplasmy; underlining indicates positions experiencing recurrent mutations within the tree while exclamation marks refer to one (!) or two (!!) back mutations relative to the RSRS. Coalescence age estimates for N1a3 and N1a3a obtained by employing the complete genome and synonymous (ρ) clocks, indicated by # and @, respectively, are also shown.

In contrast to the numerous HVS-I data, only a few complete sequences characterize the phylogeny of haplogroup N1a3 [42]. Therefore, to extend our knowledge about the phylogeography of N1a3, as well as to clarify the marker state of the C16201T substitution, we completely sequenced two N1a3 mtDNAs (one Belarusian and one Iranian Azeri), lacking the C16201T, along with 18 N1a3 samples with the classical C16201T-A16265G motif and originating from the Near/Middle East, North and South Caucasus, South, Central and East Europe. These 20 novel sequences were combined with eight mitogenomes published previously [42], [50], [51] and the resulting phylogenetic tree is shown in Figure 4.

The tree includes a major sub-branch defined by the C16201T substitution and named here as N1a3a, which encompasses most of the analyzed samples, whereas two haplotypes (Belarusian and Iranian Azeri), form two individual twigs (Figure 4). Such phylogenetic picture favors the overall stability of the C16201T transition in the phylogeny of mtDNA (www.mtdnacommunity.org and [44]), however, two independent back T16201C mutations remain a possibility.

Our wide geographic coverage of N1a3 mtDNAs identifies some features of this haplogroup. N1a3 mtDNAs from the Near/Middle East and the Caucasus include diverse set of haplotypes differing from those found in European populations. N1a3 mtDNAs from South Europe form individual limbs (Italian), but some share substitutions with the Middle Eastern and Caucasian N1a3 mtDNAs, while others – with mtDNA from central-east Europeans. N1a3 from central-east Europe encompasses both highly divergent haplotypes (Romanian and Tatar) along with almost “nodal-like” sequences (Mordvinian and Polish), characterized only by single substitutions in HVS-II. Note that central-east European N1a3 mtDNAs do not share substitutions with those from the Near and Middle East (Figure 4).

It has been suggested earlier that the Near/Middle East is most likely the region where haplogroup N1a3 has originated [42]. We found that N1a3 mtDNAs in extant human populations coalesce at 12–15 kya (Figure 4) and show distinct profiles in different geographic regions. Our data suggest that the expansion of N1a3 bearers within the Near/Middle East, Caucasus and likely in Europe took place during the Pleistocene-Holocene transition. The lack of shared N1a3 haplotypes between Near/Middle East/Caucasus and central-east Europe suggests absence of recent gene flow between these regions. It is likely that N1a3 mtDNAs experienced a period of diversification in all regions which might have been enhanced also by the low number of N1a3 bearers (either initially or decreased later).

### Phylogeny and phylogeography of mtDNA haplogroup N3

One out of 267 Belarusian mtDNAs was a member of haplogroup N3, a recently defined branch of the macro-haplogroup N (www.mtdnacommunity.org and [44]). Haplogroup N3 is characterized by the HVS-I motif T16086C, A16129G, T16172C, T16217C, G16230A, T16278C, C16311T, C16519T relative to the Reconstructed Sapiens Reference Sequence (RSRS) [51] and thus far only two N3 mitogenomes of unknown geographic origin have been reported [51]. The detection of a new basal branch of macro-haplogroup N, in particular among European populations, is a rare event, as its variation at the basal level was comprehensively characterized for this region more than a decade ago, when haplogroups R, X, I and W, which derive from haplogroup N, were defined by a combined HVS-I/RFLP (Restriction Fragment Length Polymorphism) approach [52]. Haplogroup N branches, typical of West Eurasia, have reached complete genomic characterization by now [7], [42] with four basal lineages (N1, N2, R and X), among which all but haplogroup R are, as a rule, infrequent, or, indeed, often very rare in Europe. Other N basal branches have been found elsewhere in East and South-East Asia, in Melanesia and Australia [53], [54].

The analysis of more than 30 000 HVS-I sequences including published and unpublished data shows that haplogroup N3 is extremely rare in the global human population (Figure S3, Table S6). Only 42 matching sequences have been found, suggesting an overall frequency in West Eurasia around 0.1% and even less in Europe (Figure S3). The majority of N3 mtDNAs are found in the Middle East, in agreement with an ancestral homeland in that area. N3 mtDNAs are not detected among about 2000 Africans but one Egyptian from the current study, not in the Caucasus (n = 2300), not across the Volga-Uralic region (n = 1200), Central and East Asians (n = 3500), Siberians (n = 600) and Native Americans. It was also not seen among more than 2000 subjects of South Asia either (beside one Pathan-speaking individual from Pakistan) (Figure S3, Table S6).

**Figure 5:**
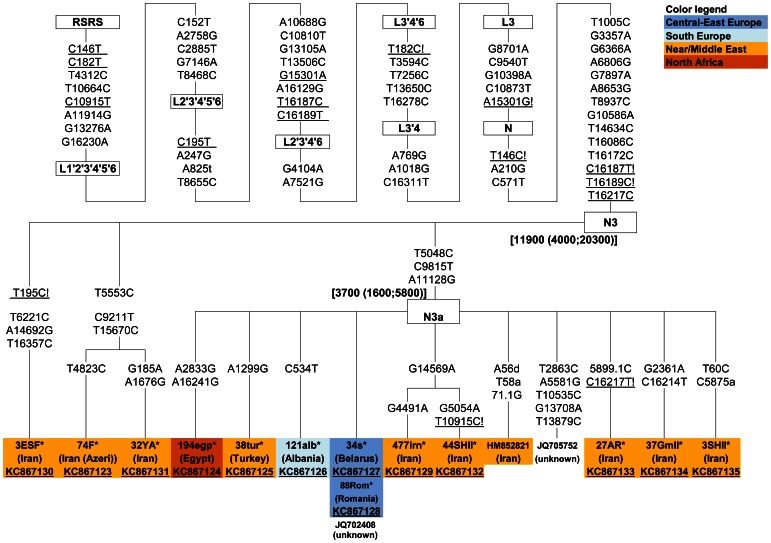
Maximum parsimony tree of mtDNA haplogroup N3. The tree includes 13 novel (marked with an asterisk and underlined accession numbers) and three previously published [50], [51] complete sequences. Mutations relative to the RSRS [51] are shown on the branches; transversions are specified with a lower case letter; underlining indicates positions which experienced recurrent mutations within the tree, while the exclamation mark (!) refers to one back mutation relative to the RSRS. Rho coalescence time estimates and their confidence intervals for haplogroup N3 and its major sub-branch N3a obtained from the complete genome clock are also shown.

To get some insight into the phylogeny and phylogeography of this novel haplogroup, we sequenced 13 N3 mitogenomes and analyzed them together with three published mtDNAs [50], [51]. The overall phylogeny of the 16 N3 mitogenomes is shown in Figure 5.

Thirteen mtDNAs form a major star-like clade within the tree, named N3a, which is defined by the motif T5048C-C9815T-A11128G. This sub-branch includes the Belarusian mtDNA and all European members of N3, together with several mtDNAs from Iran. A second sub-branch of N3, defined also by three transitions in the coding region (T5553C, C9211T, T15670C), encompasses two mtDNAs from Iran. Finally, an additional Iranian sequence forms an individual twig (Figure 5).

Considering 17 substitutions that separate the N-root from the N3-root (Figure 5), haplogroup N3 has likely originated as early as other N-branches. However, a very restricted number of its descendants are detected in extant human populations. The coalescence age of N3 mtDNAs points to an expansion at the Pleistocene-Holocene boundary (12 kya with 95% confidence intervals from 4 to 20 ky), whereas the major N3a sub-clade expanded relatively recently, likely within the last 5000 years (Figure 5).

The highest incidence and diversity of N3 mtDNAs are found in populations of present-day Iran, indicating its territory as the most likely ancestral homeland for the haplogroup. It appears that N3a has spread “successfully” in the last millennia from the Iranian area reaching North Africa in the south-west and central-east Europe in the north-west, but not the Caucasus area or central-south Asia. Taking into account the distribution pattern of N3 in the Middle East extending west to the Balkan region up to territories of Bulgaria and Romania, and its virtual absence elsewhere, it is most likely that N3 has dispersed to Europe through the Anatolian-Balkan path.

Although the spread patterns of haplogroups N1a3 and N3 bear obvious similarities (Figures 4 and 5), it is worthwhile to notice some of the differences. While the former is well spread all over the western Asia – in Iran as well as in Arab-speaking areas and in the Caucasus, the latter is largely restricted to Iran. It suggests that movement of populations over the West Asian space during the Pleistocene-Holocene boundary, coinciding perhaps with the end of the Younger Dryas, was more intense than in later periods, during the mid-Holocene, well after the transition of human to agriculture and largely sedentary lifestyle.

### NRY profile of Belarusians

**Figure 6:**
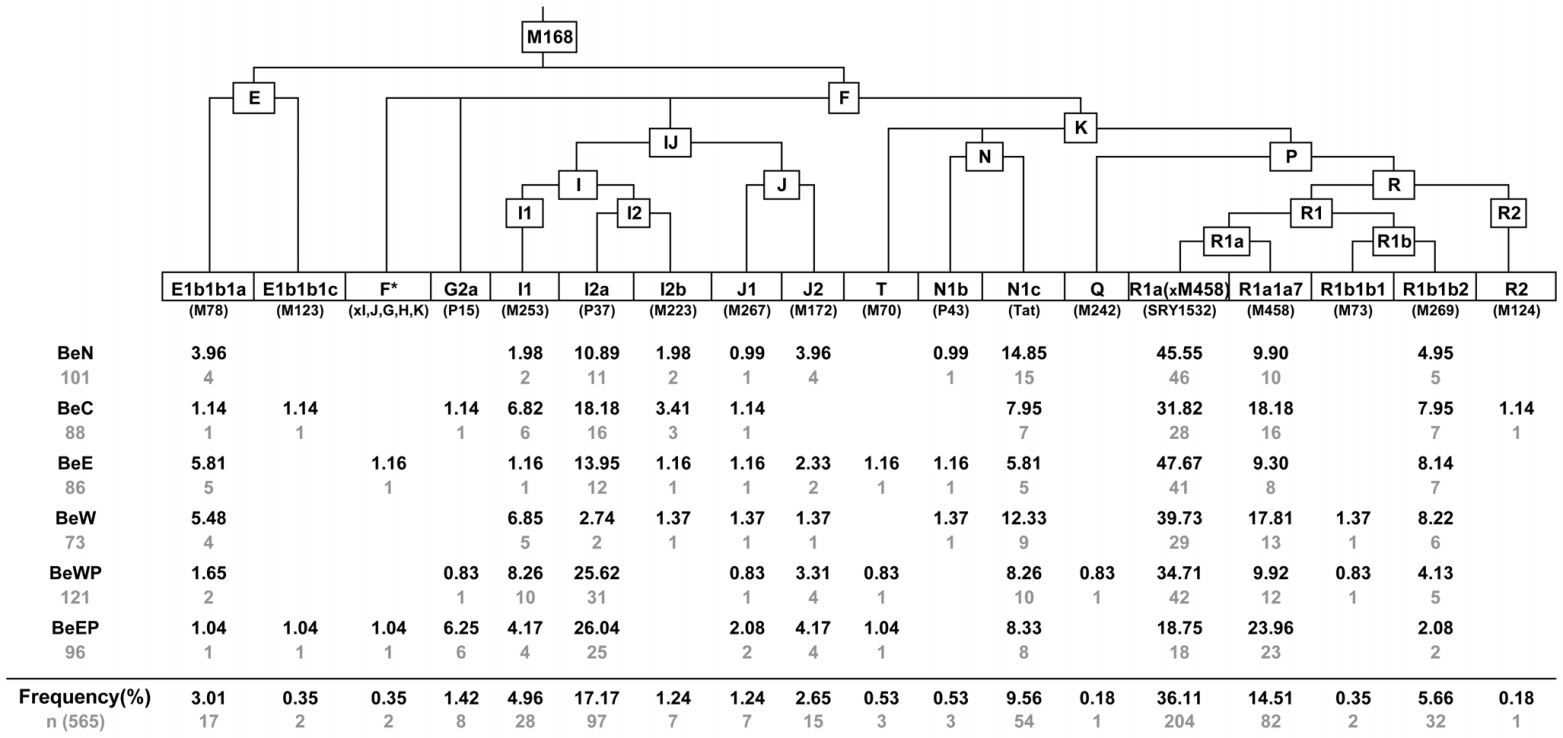
Phylogeny of NRY haplogroups and their relative frequencies in Belarusians. Haplogroup-defining biallelic markers are in parentheses. Belarusian sub-populations are designated as BeN – North, BeC – Centre, BeE – East, BeW – West, BeWP – West Polesie, BeEP – East Polesie. Sample sizes and absolute frequencies are also given.

More than one half of Belarusian males belong to the haplogroup R1a(SRY1532), which together with I2a(P37) and N1c(Tat) NRY lineages cover almost 80% of the NRY diversity of population; the rest is represented by numerous less frequent haplogroups and sub-haplogroups (Figure 6). A substantial portion of R1a chromosomes, bearing the derived M458G allele, have an indigenous European origin [55]. In Belarusians, another branch of haplogroup R, haplogroup R1b(M269), makes up around 5% (Figure 6). It was shown previously [56] that this lineage encompasses several sub-branches (i.e. East European-, Caucasus- and south Siberian-specific) among eastern Europeans including Belarusian population. Haplogroup I in Belarusians is composed of multiple genetic inputs, mainly from the north-western Balkans (I2a(P37)), and, to a lesser extent, from West and north-west Europe (I1(M253), I2b(M223)) [57]. N1c(Tat) along with its much less frequent sister group N1b(P43) (previously N2), detected in Belarusians indicate an ancient patrilineal gene flow from the north Eurasia westward, yet in the context of studied here populations is best explained by partially shared Y-chromosomal ancestry of Belarusians and their northern neighbors, Lithuanians and Latvians, among whom N1c(Tat) reaches frequencies above 40% [20], [21], [26], [58]. The STR haplotypes of N1b(P43) Y-chromosomes belong to both ‘European’ and ‘Asian’ sub-clusters [58], which may indicate their different sources in Belarusians (Table S7), whereas in general we note that Belarusian population represents the westernmost fringe of N1b(P43) haplogroup distribution detected to date. J2(M172) and E1b1b1a(M78) NRY haplogroups, found in Belarusians at low frequencies, are generally typical for Anatolia and the southern Balkans [59], [60] as well as for the Caucasus region (G2a) [61], [62].

Certain NRY haplogroups show gradient-like patterns in their frequency distribution in Belarus. For example, haplogroup I2a(P37) makes up a quarter of the Y-chromosome pool in the south regions (West and East Polesie), but decreases northward, in agreement with the earlier observed south-west – north-east spread of this haplogroup [57]. Contrary to that, haplogroup N1c(Tat) shows the highest frequency (around 15%) in north-west Belarus and is decreasing southward, as it could be expected, bearing in mind that among Lithuanians N1c(Tat) comprises close to a half of their Y-chromosomes [21]. Haplogroup R1a(SRY1532) has slightly lower frequencies in West and East Polesie compared with the rest of Belarus. The share of R1a1a7(M458) Y-chromosomes *vice versa* decreases from south-west toward north and east of Belarus when Polesie is considered as one region. To test patterns of spatial distribution of the three major NRY haplogroups (N1c(Tat), I2a(P37) and R1a(SRY1532)) in Belarusians, spatial autocorrelation analysis was performed. The correlograms show that N1c(Tat) and I2a(P37) haplogroup frequency gradients within the Belarusian region are not statistically significant likely due to a small number of points and a rather small geographic area, whereas haplogroup R1a(SRY1532) demonstrates no regular pattern (Figure S4). However we note that both N1c(Tat) and I2a(P37) NRY haplogroups demonstrate statistically significant south-north gradients within a wider Eastern European area [15].

To evaluate the intra-population structure of the paternal gene pool, a Multidimensional Scaling (MDS) analysis based on pairwise Rst values calculated from 13 Y-STRs among six sub-populations was performed (Figure S5). Our analysis revealed that south-central Belarus (West, East Polesie and Centre sub-populations) is separated from the north-western regions (BeN and BeW) on the plot, whereas the East sub-population positions apart. Similarly, pairwise Fst values, calculated from NRY haplogroup frequencies, indicate that both West and East Polesie (southern Belarus) are differentiated from other sub-regions except the Centre (BeC) (Table S8). We applied AMOVA to test the distribution of genetic variance for the same geographic subdivisions of Belarusians used in case of mtDNA data (see the Methods section for grouping details in AMOVA analysis). Analysis shows that the genetic differentiation between southern and the rest of the sub-groups is more informative (1.9% between group variation, although statistically insignificant) in comparison to western *vs* the rest of the sub-groups (Table S4). Taken together, differences of NRY gene pool within Belarus are more pronounced along its south-to-north axis than between its western and eastern regions. It has been shown that the same trend of south-north differentiation of the paternal gene pool extends eastward, encompassing sub-populations of the so-called “historical Russian area” [15], whereas it becomes less notable or even transforms into a west-east distinction further westward [23].

Similarly to other East Slavic-speakers, almost a third of the Belarusian paternal gene pool is constituted of two haplogroups, I2a(P37) and N1c(Tat). The first indicates gene flow from south-east Europe northward [57], while N1c(Tat) is largely spread among north Eurasians and within central-east Europe reaches the Ukrainians in the south, though only marginally, the Poles in the west [58]. To determine the relationship of Y-STR haplotypes between the populations of East Europe and the Balkans and also to get an idea about the origin of the two haplogroups in the extant Belarusian population, we have calculated their Median-Joining (MJ) networks (Table S9).

**Figure 7:**
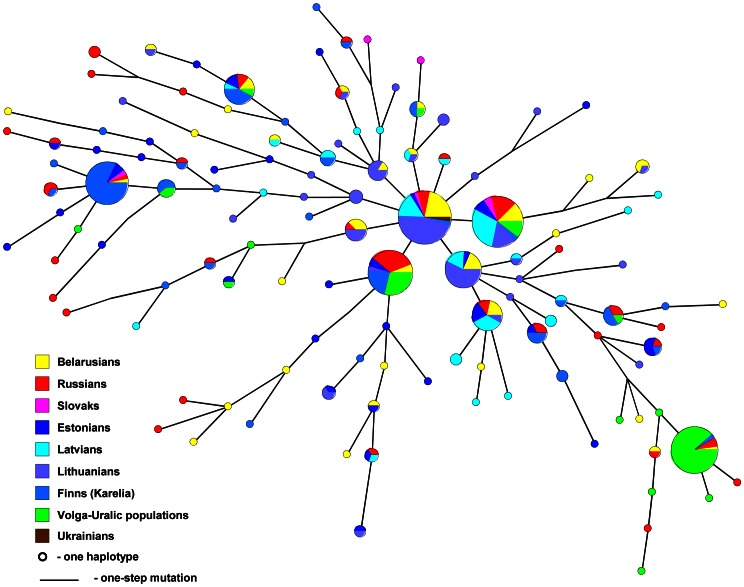
Maximum Parsimony tree (based on MJ network) of NRY haplogroup N1c(Tat) calculated from *seven* Y-STRs. Volga-Uralic populations include Komis (Priluzhski, Izhevski), Udmurts, Maris, Bashkirs, Chuvashes. Altogether 402 individuals are analyzed, the sample size of each population and the set of Y-STRs used for calculations are given in Table S9.

Maximum Parsimony (MP) tree (based on MJ network) of haplogroup N1c(Tat) includes West and East Slavic-speakers, Balts, Estonians, Finns (from the south and north Karelian regions of Finland) and populations of the Volga-Uralic region (Komis, Udmurts, Maris, Chuvashes and Bashkirs) (Figure 7). N1c(Tat) haplotypes tend to show regional specificity within East Europe. Three groups of haplotypes can be distinguished based on their prevalence among certain populations: the ones, most common among the Volga-Uralic populations; those spread primarily among Finns and N1c(Tat) haplotypes that are found largely among Balts, and Slavic-speakers. It has been suggested that the differentiation of N1c(Tat) haplotypes between Balts and the Volga-Uralic populations was due to the splitting of a founder on the way of its migration towards the Baltic Sea region and was strengthened by genetic drift [20], [22]. The N1c(Tat) tree in this study indicates that Belarusians share a considerable portion of haplotypes with Balts pointing to a shared patrilineal founder(s) and history. Beside this, Belarusian N1c(Tat) encompasses haplotypes both individual ones and those, shared with Finns as well as with the Volga-Uralic populations (Figure 7). Hence, it is possible that in addition to the suggested split of the N1c(Tat) founder(s) during its spread westward, the diversity of haplogroup N1c(Tat) as observed in Belarusians, could have been shaped by reciprocal movements of its bearers within East Europe.

**Figure 8:**
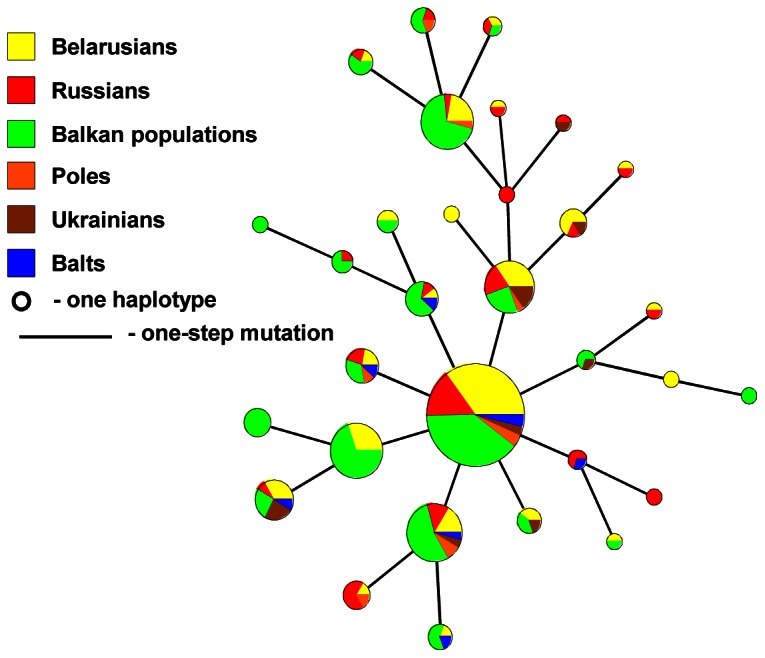
Maximum Parsimony tree (based on MJ network) of NRY haplogroup I2a(P37) calculated from *seven* Y-STRs. Balkan populations include Bosnians, Croatians and Slovenians. Altogether 347 individuals are analyzed, the sample size of each population and the set of Y-STRs used for calculations are given in Table S9.

Unlike haplogroup N1c(Tat), the microsatellite haplotypes of haplogroup I2a(P37) of Balts, East, West Slavic populations and Balkan peoples (Bosnians, Croats and Slovenians) show a star-like branching pattern (Figure 8). Furthermore, most of the I2a(P37)-haplotypes analyzed are shared among populations inhabiting a wide geographic area.

Thus, the analyses reveal similar I2a(P37)-founder(s) for Belarusians and Balkan populations, whereas haplogroup N1c(Tat) in Belarusians is an assemblage of largely “Balto-Slavic” specific haplotypes along with those spread in Volga-Uralic and Finnic populations.

**Figure 9:**
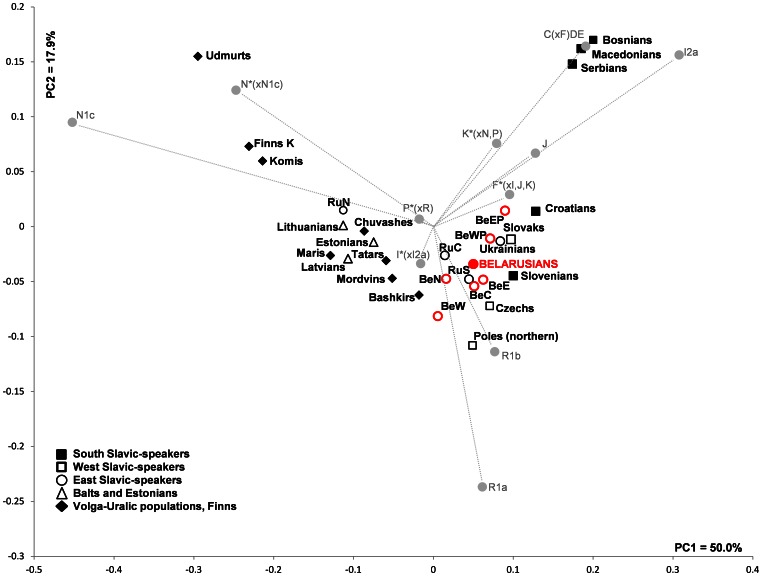
PC plot based on NRY haplogroup frequencies among eastern Europeans and Balkan populations. The contribution of each haplogroup to the first and the second PCs are shown in gray. Population abbreviations are as follows: BeN, BeW, BeC, BeWP, BeEP, BeE – Belarusians from North, West, Central, West Polesie, East Polesie and East sub-regions, respectively, filled red circle denotes the total Belarusian population; RuS, RuC, RuN – Russians from southern, central and northern regions, respectively; Finns K – Finns from Karelia. K*(x N,P) refers to samples with M9, M20, M70 derived alleles and 92R7, M214 ancestral alleles; P*(xR) refers to samples with 92R7, M242 derived alleles and M207 ancestral allele; F*(xI,J,K) refers to samples with M89 derived allele and M9, M201, M170, 12f2 ancestral alleles; C(xF)DE refers to samples with Yap and M130 derived and M89 ancestral alleles. Frequencies of NRY haplogroups and references are listed in Table S3.

The PC plot based on the frequencies of NRY haplogroups (Figure 9) assesses the relationships between paternal gene pools of Belarusians and other eastern European and Balkan populations speaking Slavic, Baltic, Finno-Permic and Turkic languages. Belarusians, Russians and Ukrainians, the three East Slavic-speakers and geographic neighbors, are the closest according to their patrilineal legacy. We also note that southern sub-populations of Belarus group with Ukrainians whereas northern and western ones are moved toward Volga-Uralic populations (Figure 9) - the intra-population pattern that was observed also in the MDS plot based on Y-STRs (Figure S5). Two West Slavic-speaking populations (Czechs and Poles) are shifted from East Slavs due to higher frequencies of R1b and R1a NRY haplogroups, respectively (Table S3), whereas Slovaks remain close to Eastern Slavic group. Among the South Slavic-speakers, Slovenians and Croatians stay closer to East Slavic-speakers, while Bosnians, Macedonians and Serbians form a distant group, mainly due to high frequencies of haplogroups I2a(P37) and E (Table S3). Balts, Estonians and Volga-Uralic populations, who are northern and eastern neighbors of the Slavic-speakers, stay also apart due to considerably different frequencies of NRY haplogroups observed in East Slavic-speakers, that is, a prevalence of haplogroup N1c(Tat), a decreased R1a(SRY1532) and minute frequencies of I2a(P37) (Figure 9, Table S3). Hereby, PC analysis reveals that the NRY pool of central and eastern Europeans and Balkan populations harbors a marked geographic structure, whereas Belarusians, Ukrainians and Russians (except northern regions of the latter) tend to cluster in agreement with their common East Slavic linguistic affiliation and previously published observations [15].

### The intra-population structuring of Belarusians

The watershed between the Baltic Sea and the Black Sea divides Belarus roughly into north-eastern (rivers descending towards the Baltic Sea basin) and south-western (rivers descending towards the Black Sea) regions. The latter, known as Polesie, is vast lowland rich in swamps and forests, and differs markedly from the northern region of Belarus. Rural populations all over Belarus, non-uniform in physical characteristics, speak numerous dialects, as an outcome of a long-lasting sedentary agricultural lifestyle [63]–[65]. Yet the pattern of their genetic variation does not follow only isolation-by-distance differentiation caused by random genetic drift. As it was pointed above, these largely latitudinal gradients within the Belarus people, reflect likely ancient movements of Y-chromosomes (males) from north to south, as testified by the spread pattern of N1c(Tat)-chromosomes with their frequency peak around 50–70% in eastern Fennoscandia [26], [27], [58], and, secondly, the northward movements of the carriers of NRY haplogroup I2a(P37), that have likely originated from north-western Balkans [57], [60]. The longitudinal Y-chromosomal intra-Belarus variation is less pronounced (Table S4) likely because the present-day Belarus area lies within the very epicenter of the initial spread of the dominant R1a (among the Belarus population haplogroup), including its R1a1a7(M458) limb, around the Pleistocene-Holocene boundary [55]. Because the spread of I2a(P37) Y-chromosomes from the Balkans, as well as the expansion of N1c(Tat) in East Europe, have been dated to early Holocene [26], [60], one may conclude that the core of the Belarusian patrilineal pool, comprising about three quarters of its present-day variation, may have been formed during the post-Younger Dryas - early Holocene period.

Our mtDNA data, on the other hand, suggest that genetic variation between sub-populations in Belarusians is low. Whereas PC and AMOVA analyses indicate a slight difference between southern Belarusians and the rest of the sub-populations (Figure S1, Table S4), pairwise population Fst values reveal very little or no genetic differentiation (Table S5). Thereby, much larger and deeper (preferentially complete mtDNA genomic level) studies of Belarusians and their neighboring populations would be needed to reliably reveal the potential shared ancestry and matrilineal gene flows in the region.

It is also worth noting that high-density whole genome studies [66] show that the genetic structure of Belarusians, similarly to that of their immediate neighbors, largely comprises of two major ancestry components spread across the north-eastern and southern European regions with marginal East Eurasian-specific contribution.

To sum up, the phylogeography of the Belarusian patrilineal heritage, although overwhelmingly West Eurasian by descent and dominated by R1a, the most prevalent haplogroup among West and East Slavs, reveals two latitudinal gradients, reflecting prehistoric and historic time admixture with the Baltic-speaking people in the north (haplogroup N1c) and gene flow from the north-western Balkans (haplogroup I2a). Meanwhile, East Eurasian Y-chromosomal (Q) and mitochondrial DNA (M including C, D, G) variants are very rare among Belarusians. Detecting a new basal branch of a macro-haplogroup N – haplogroup N3 – among Belarusians, came as a surprise and provoked its further study, alongside with somewhat less, but still rare haplogroup N1a3. Mainly Middle Eastern, the phylogeography of haplogroup N3 may represent a detectable gene flow from Middle East to Europe during mid-Holocene. In contrast to N3, the phylogeography of haplogroup N1a3 and its major sub-clade N1a3a, particularly, demonstrates a high diversity over a wide geographic area covering Middle East, Caucasus and Europe, and attests a significantly earlier expansion of its bearers, likely, around the Pleistocene-Holocene transition.

## Methods

### Samples

For sampling reasons the territory of Belarus was divided into six geographic sub-regions: North (West Dvina River region), East (Dnieper River region), West (Neman River region), West Polesie (south-west region of Belarus), East Polesie (south-eastern region of Belarus) and Centre, the latter located in between the other five (Figure 1). To avoid demographic effects due to the last century industrial urbanization, sampling was carried out only in small towns and villages. Altogether 565 Y-chromosomes and 267 mtDNAs of ethnic Belarusians were analyzed. The regions and sample sizes are listed in Table S1.

All volunteers filled a detailed questionnaire ascertaining the ethnicity and birth-place of themselves as well as their parents and grandparents. Only adult volunteers, who resided in the region of interest and whose ancestors had lived there for the last three generations, were included in the study. Intra-venous blood was collected from healthy unrelated males. DNA samples were obtained using the standard protocol with proteinase K following phenol-chloroform extraction and ethanol precipitation [67].

### Ethics Statement

The population samples analyzed in the study were collected after having obtained a written informed consent. The study has been considered and approved specifically by the Bioethics Committee of the Belarusian State Medical University (Minsk, Belarus) and Scientific Boards of the participating research institutions.

### Genotyping

Y-chromosome data were generated by genotyping (RFLP or direct sequencing) 28 single nucleotide polymorphisms (SNPs) and *indels* (insertions, deletions) (M89, Yap, M35, M78, M123, M201, P15, M170, M253, P37, M223, 12f2/SRY, M267, M172, M9, M70, M231, P43, Tat, 92R7, M207, M173, SRY1532, M458, M73, M269, M124, M242) in 565 samples according to the current Y-chromosome phylogeny [55], [68]. Note, the following markers: M174 (haplogroup D), M130 (haplogroup C), M81 (within haplogroup E), M22 (haplogroup L) and M82 (haplogroup H) were typed but not observed. In total, 17 NRY haplogroups were inferred whereas two samples remained in a paragroup (F*(x I, J, G, H, K)). Additionally, 14 Y-STRs were genotyped in all samples (DYS19, DYS385ab, DYS389I,II, DYS390, DYS391, DYS392, DYS393, DYS437, DYS438, DYS439, DYS448, DYS456, DYS458 and H4).

For mtDNA, HVS-I from nucleotide positions 16000 to 16400 was sequenced in 267 Belarusian samples. Complete mtDNA sequencing was performed for 33 samples in total, in part according to [69], in part applying the methodology described in [70]. Sequences were aligned and analyzed by using ChromasPro version 1.5 (Technelysium Pty Ltd), and nucleotide mutations were initially ascertained relative to the revised Cambridge Reference Sequence (rCRS) [71]. Then, in order to record HVS-I and complete mitogenome polymorphic positions relative to the RSRS [51], the FASTmtDNA utility provided by MtDNA Community (www.mtdnacommunity.org) was applied. HVS-I and coding-region substitutions (Table S10) were used to resolve haplogroup status following the hierarchy of the mtDNA phylogenetic tree (www.mtdnacommunity.org and [44]). MtDNA haplogroups were designated according to the current nomenclature; transitions, transversions, back mutations were labeled following the established style (www.mtdnacommunity.org and [44]). Polymorphic nucleotide positions recorded relative to the RSRS and rCRS for 33 completely sequenced mtDNAs in this study are listed in Table S11.

### Data analysis

Y-STR haplotype phylogenies for major NRY haplogroups in the Belarusian population were constructed using Network 4.6.0.0, applying the MJ algorithm (Fluxus Technology Ltd, http://fluxus-technology.com). Weights of loci were chosen according to their variability, post-processing MP calculations were performed and MP trees of NRY haplogroups were drawn using Network Publisher [72]. DYS385 was excluded from all further calculations, DYS389I was subtracted from DYS389II and both were included in the calculations. When data from reference populations were included in the analysis of NRY haplogroup phylogenies, the restricted available set of Y-STRs was used (specified for each haplogroup in Table S9). The Y-STR haplotypes for the N1c(Tat), N1b(P43) and I2a(P37) NRY haplogroups of Belarusians are listed in Table S7 and Table S12, respectively.

Arlequin 3.5 software [73] was used to calculate genetic distance indices (Rst, Fst) and to assess the genetic structure in Belarusians by AMOVA. Two major geographical subdivisions of Belarusians were considered in AMOVA: (a) southern (West and East Polesie) *vs* the remaining four sub-populations (Centre, West, East and North) and (b) western (West and West Polesie) *vs* the remaining four sub-populations (Centre, East Polesie, East and North). MDS was performed using Statistica 6.0 Software (http://www.statsoft.com). PC analysis was performed using the *popstr* algorithm (http://harpending.humanevo.utah.edu/popstr/). Frequencies of mtDNA and NRY haplogroups used in the PC analyses are listed in Table S3. To test patterns of spatial distribution of the three major NRY haplogroups in Belarusians (N1c(Tat), I2a(P37) and R1a(SRY1532)), Moran's I autocorrelation coefficients were calculated using binary weight matrix with five distance classes and random distribution assumption using in the PASSAGE software V.1.1 (release 3.4) (http://www.passagesoftware.net/) [74]. Note that six Belarusian sub-populations (Table S1) together with immediate neighbors (Poles, Lithuanians, Latvians, Central Russians and Ukrainians) were included in the analysis.

To estimate the age of mtDNA lineages, we calculated rho-statistics (ρ) as average number of substitutions from the root haplotype [75] and its standard deviation (σ) [76]. The calculator provided by [77] was used to convert the ρ-statistics and its error ranges to age estimates with 95% confidence intervals.

## Supporting Information

Figure S1 **PC analysis based on mtDNA haplogroup frequencies in six Belarusian sub-populations.** The distribution of the populations within 1–2 and 1–3 PCs is represented in the upper panels; the contribution of mtDNA haplogroups to each of the PCs is depicted in the lower panels. Sub-populations are designated as BeN – North, BeC – Centre, BeE – East, BeW – West, BeWP – West Polesie, BeEP – East Polesie.
(DOCX)

Figure S2 **PC analysis based on mtDNA haplogroup frequencies among eastern Europeans and Balkan populations.** The contribution of each haplogroup to the first and the second PCs is shown in gray. The group “Other” includes “Other” from published data merged with uncommon haplogroups L1b, L2a and L3f. Frequencies of mtDNA haplogroups and references are listed in Table S3.
(DOCX)

Figure S3 **The distribution of mtDNA haplogroup N3 in world populations retrieved from published data along with those generated in this study.** Black squares refer to the screened population data; green circles mark regions where haplogroup N3 has been detected and a star sign denotes the geographic origin of N3 mtDNAs completely sequenced in this study. Numbers inside the star correspond to the number of mtDNAs. See Table S6 for reference data and number of N3 sequences detected in each population.
(DOCX)

Figure S4 **Spatial autocorrelation analysis for three major NRY haplogroups (N1c(Tat), I2a(P37) and R1a(SRY1532)) in Belarusians.** Moran's I indices were calculated for three NRY haplogroups in six Belarusian sub-populations including also immediate neighbor populations (Ukraine, Poland, Lithuania, Latvia, Central Russia). Correlograms indicate that ‘gradient-like’ frequency patterns for N1c(Tat) and I2a(P37) haplogroups are not statistically supported due to likely small number of points and rather small geographic area. Haplogroup R1a(SRY1532) demonstrates no pattern in its frequency distribution. Open circles in correlograms denote non-significant values.
(DOCX)

Figure S5 **MDS plot of pair-wise Rst values obtained from 13 Y-STRs in the six Belarusian sub-populations (stress = 0.0000048).** Sub-populations are designated as follows: BeN – North, BeC – Centre, BeE – East, BeW – West, BeWP – West Polesie, BeEP – East Polesie.
(DOCX)

Table S1 **Geographic origin of the Belarusian samples.**
(XLSX)

Table S2 **MtDNA control and coding region polymorphisms in Belarusians.**
(XLSX)

Table S3 **MtDNA and NRY reference data used in PC analyses.**
(XLS)

Table S4 **Analysis of molecular variance in Belarusians.**
(DOCX)

Table S5 **Pairwise population Fst calculated from mtDNA haplogroup frequencies in six Belarusian sub-populations.**
(DOCX)

Table S6 **The distribution of N3 mtDNAs (T16086C, A16129G, T16172C, T16217C, G16230A, T16278C, C16311T) in world populations.**
(XLSX)

Table S7 **Y-chromosome N1c(Tat) and N1b(P43) STR haplotypes in Belarusians.**
(XLSX)

Table S8 **Pairwise population Fst calculated from NRY haplogroup frequencies in six Belarusian sub-populations.**
(DOCX)

Table S9 **Y-STR data used in Median-Joining Networks calculations.**
(XLSX)

Table S10 **MtDNA haplogroups defining control (HVS-I: 16000–16400) and coding-region mutations relative to the RSRS and rCRS.**
(XLSX)

Table S11 **Complete haplotypes of N3 and N1a3 mtDNAs generated in this study.**
(XLSX)

Table S12 **Y-chromosome I2a(P37) STR haplotypes in Belarusians.**
(XLSX)
