# Supplementary material for: Uniparental Genetic Heritage of Belarusians: Encounter of Rare Middle Eastern Matrilineages with a Central European Mitochondrial DNA Pool
Source: PLoS One. 2013 Jun 13;8(6):e66499. doi: 10.1371/journal.pone.0066499 (PMC3681942; doi:10.1371/journal.pone.0066499)
Supplement: Figure S1 — PC analysis based on mtDNA haplogroup frequencies in six Belarusian sub-populations. The distribution of the populations within 1–2 and 1–3 PCs is represented in the upper panels; the contribution of mtDNA haplogroups to each of the PCs is depicted in the lower panels. Sub-populations are designated as BeN – North, BeC – Centre, BeE – East, BeW – West, BeWP – West Polesie, BeEP – East Polesie. (DOCX) [file pone.0066499.s001.docx]

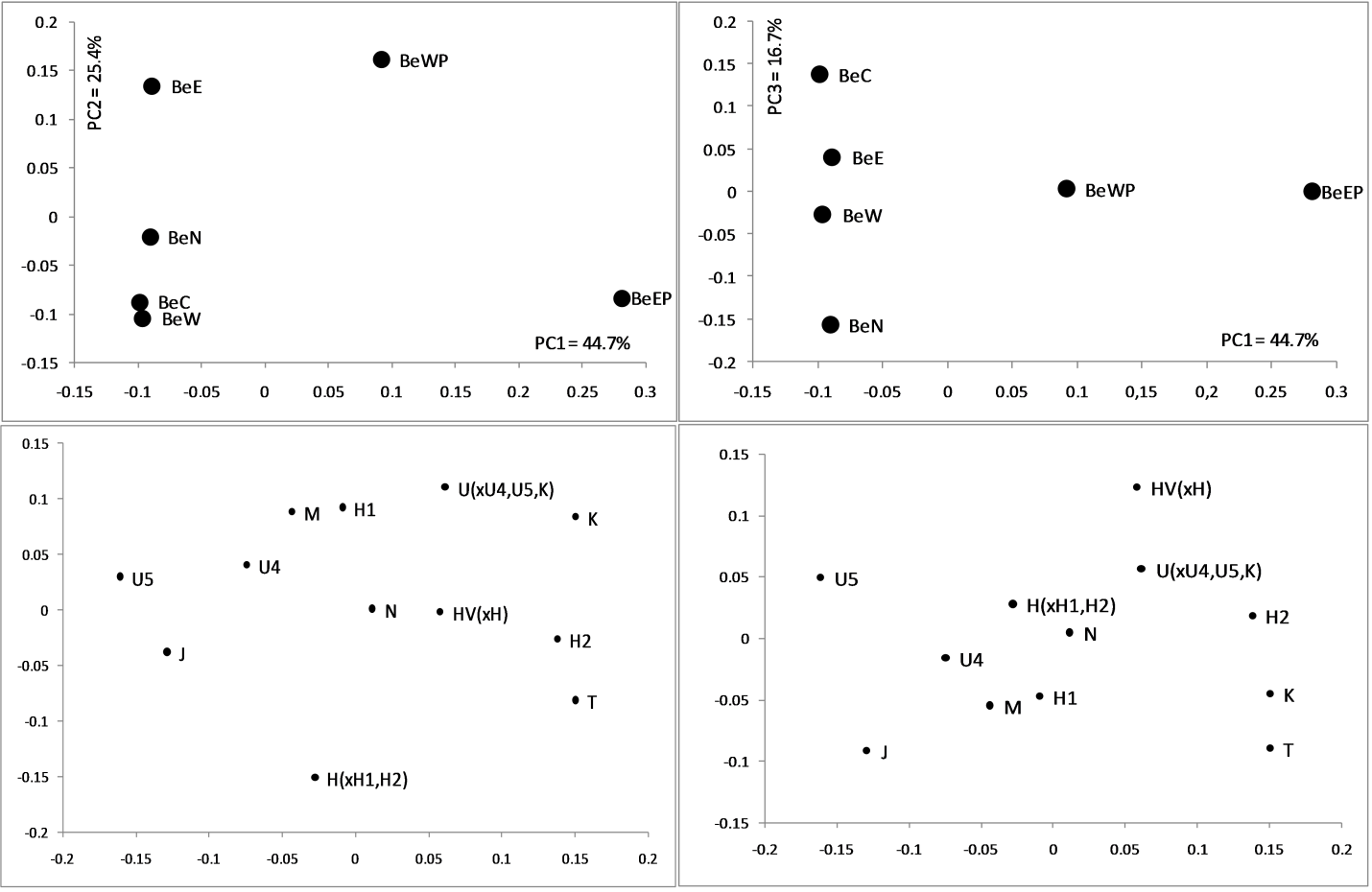


**Figure S1. PC analysis based on mtDNA haplogroup frequencies in six Belarusian sub-populations.** The distribution of the populations within 1-2 and 1-3 PCs is represented in the upper panels; the contribution of mtDNA haplogroups to each of the PCs is depicted in the lower panels. Sub-populations are designated as BeN – North, BeC – Centre, BeE – East, BeW – West, BeWP – West Polesie, BeEP – East Polesie.
