# Supplementary material for: Uniparental Genetic Heritage of Belarusians: Encounter of Rare Middle Eastern Matrilineages with a Central European Mitochondrial DNA Pool
Source: PLoS One. 2013 Jun 13;8(6):e66499. doi: 10.1371/journal.pone.0066499 (PMC3681942; doi:10.1371/journal.pone.0066499)
Supplement: Figure S2 — PC analysis based on mtDNA haplogroup frequencies among eastern Europeans and Balkan populations. The contribution of each haplogroup to the first and the second PCs is shown in gray. The group “Other” includes “Other” from published data merged with uncommon haplogroups L1b, L2a and L3f. Frequencies of mtDNA haplogroups and references are listed in Table S3. (DOCX) [file pone.0066499.s002.docx]

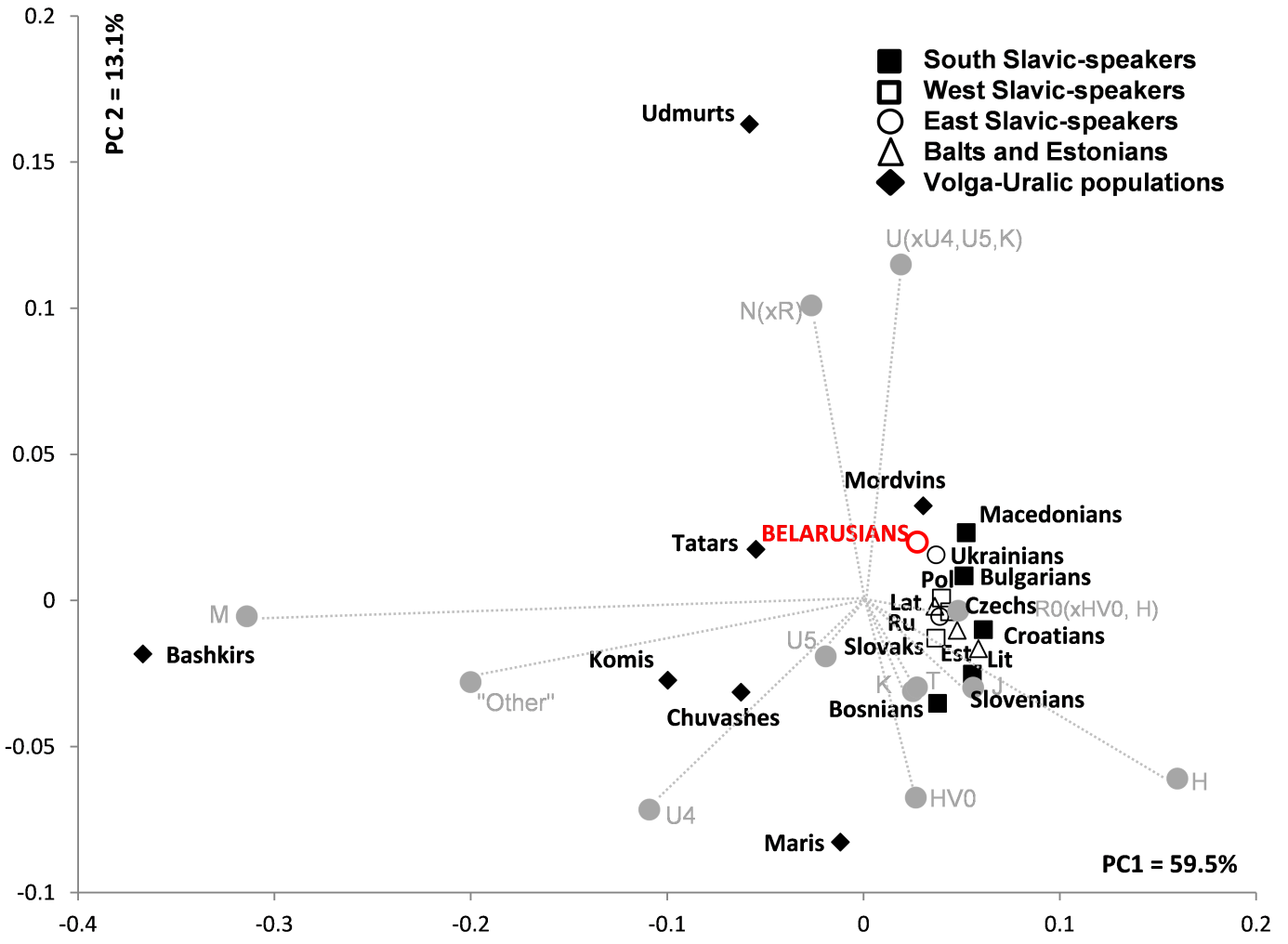


**Figure S2. PC analysis based on mtDNA haplogroup frequencies among eastern Europeans and Balkan populations.** The contribution of each haplogroup to the first and the second PCs is shown in gray. The group “Other” includes “Other” from published data merged with uncommon haplogroups L1b, L2a and L3f. Frequencies of mtDNA haplogroups and references are listed in Table S3.
