# Supplementary material for: Uniparental Genetic Heritage of Belarusians: Encounter of Rare Middle Eastern Matrilineages with a Central European Mitochondrial DNA Pool
Source: PLoS One. 2013 Jun 13;8(6):e66499. doi: 10.1371/journal.pone.0066499 (PMC3681942; doi:10.1371/journal.pone.0066499)
Supplement: Figure S3 — The distribution of mtDNA haplogroup N3 in world populations retrieved from published data along with those generated in this study. Black squares refer to the screened population data; green circles mark regions where haplogroup N3 has been detected and a star sign denotes the geographic origin of N3 mtDNAs completely sequenced in this study. Numbers inside the star correspond to the number of mtDNAs. See Table S6 for reference data and number of N3 sequences detected in each population. (DOCX) [file pone.0066499.s003.docx]

**
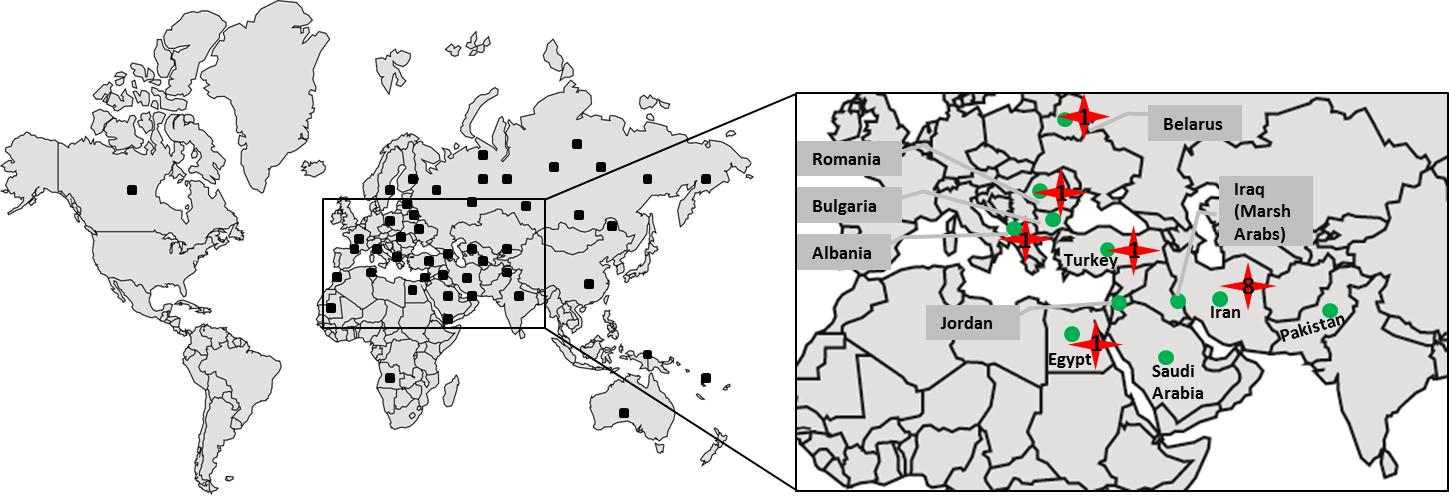
**

**Figure S3. The distribution of mtDNA haplogroup N3 in world populations retrieved from published data along with those generated in this study.** **Black squares** refer to the screened population data; **green circles** mark regions where haplogroup N3 has been detected and a **star** sign denotes the geographic origin of N3 mtDNAs completely sequenced in this study. Numbers inside the star correspond to the number of mtDNAs. See Table S6 for reference data and number of N3 sequences detected in each population.
