# Supplementary material for: Uniparental Genetic Heritage of Belarusians: Encounter of Rare Middle Eastern Matrilineages with a Central European Mitochondrial DNA Pool
Source: PLoS One. 2013 Jun 13;8(6):e66499. doi: 10.1371/journal.pone.0066499 (PMC3681942; doi:10.1371/journal.pone.0066499)
Supplement: Figure S4 — Spatial autocorrelation analysis for three major NRY haplogroups (N1c(Tat), I2a(P37) and R1a(SRY1532)) in Belarusians. Moran's I indices were calculated for three NRY haplogroups in six Belarusian sub-populations including also immediate neighbor populations (Ukraine, Poland, Lithuania, Latvia, Central Russia). Correlograms indicate that ‘gradient-like’ frequency patterns for N1c(Tat) and I2a(P37) haplogroups are not statistically supported due to likely small number of points and rather small geographic area. Haplogroup R1a(SRY1532) demonstrates no pattern in its frequency distribution. Open circles in correlograms denote non-significant values. (DOCX) [file pone.0066499.s004.docx]

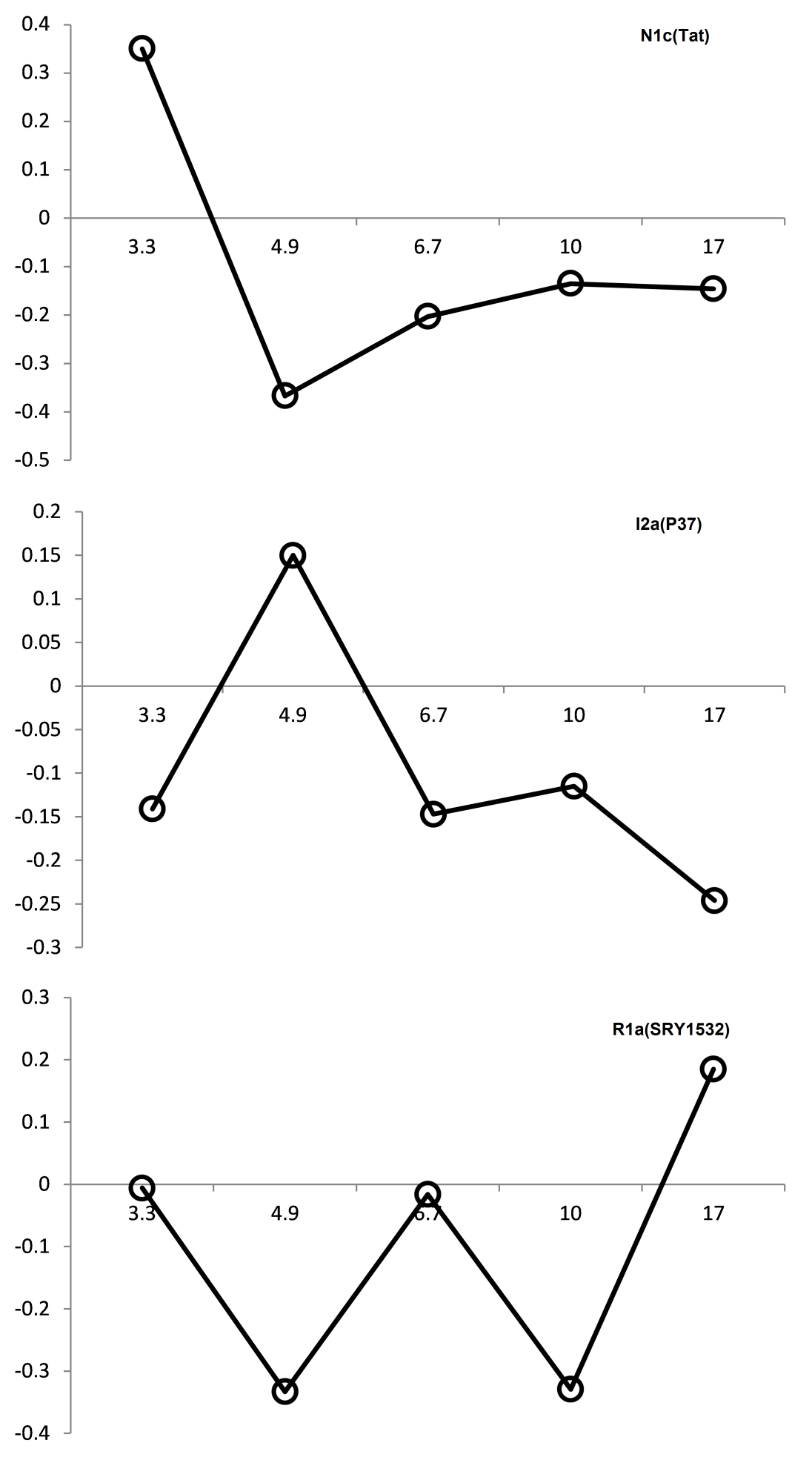


**Figure S4. Spatial autocorrelation analysis for the three major NRY haplogroups (N1c(Tat), I2a(P37) and R1a(SRY1532)) in Belarusians.** Moran’s I indices were calculated for the three NRY haplogroups in six Belarusian sub-populations including also immediate neighbor populations - Ukraine, Poland, Lithuania, Latvia and Central Russia - to assure that the territory of Belarus is covered completely. The correlograms indicate that ‘gradient-like’ frequency patterns for N1c(Tat) and I2a(P37) haplogroups are not statistically supported due to likely small number of points and a rather small geographic area. Haplogroup R1a(SRY1532) demonstrates no pattern in its frequency distribution. Open circles in the correlograms denote non-significant values.
