# Supplementary material for: Uniparental Genetic Heritage of Belarusians: Encounter of Rare Middle Eastern Matrilineages with a Central European Mitochondrial DNA Pool
Source: PLoS One. 2013 Jun 13;8(6):e66499. doi: 10.1371/journal.pone.0066499 (PMC3681942; doi:10.1371/journal.pone.0066499)
Supplement: Figure S5 — MDS plot of pair-wise Rst values obtained from 13 Y-STRs in the six Belarusian sub-populations (stress = 0.0000048). Sub-populations are designated as follows: BeN – North, BeC – Centre, BeE – East, BeW – West, BeWP – West Polesie, BeEP – East Polesie. (DOCX) [file pone.0066499.s005.docx]

**Figure S5. MDS plot of pair-wise Rst values obtained from 13 Y-STRs in the six Belarusian sub-populations (stress=0.0000048).** Sub-populations are designated as follows: BeN – North, BeC – Centre, BeE – East, BeW – West, BeWP – West Polesie, BeEP – East Polesie.
