# Supplementary material for: Uniparental Genetic Heritage of Belarusians: Encounter of Rare Middle Eastern Matrilineages with a Central European Mitochondrial DNA Pool
Source: PLoS One. 2013 Jun 13;8(6):e66499. doi: 10.1371/journal.pone.0066499 (PMC3681942; doi:10.1371/journal.pone.0066499)
Supplement: Table S4 — Analysis of molecular variance in Belarusians. (DOCX) [file pone.0066499.s009.docx]

**Table S4**. Analysis of molecular variance in Belarusians.

|  | **MtDNA** | |  | **NRY** | |
| --- | --- | --- | --- | --- | --- |
|  | ^a^Southern *vs* rest | ^b^Western *vs* rest |  | ^a^Southern *vs* rest | ^b^Western *vs* rest |
|  |  |  |  |  |  |
| Among groups | 0.32** | -0,51 |  | 1.9 | -1.4 |
|  |  |  |  |  |  |
| Among populations within groups | 0.27 | 0.69* |  | 1.2* | 3.08* |
|  |  |  |  |  |  |
| Within populations | 99.41* | 99.82 |  | 96.9* | 98.32* |
|  |  |  |  |  |  |

^a^ – West Polesie, East Polesie (southern Belarus) *vs* Centre, West, East, North (the remaining four populations);

^b^ – West and West Polesie (western Belarus) *vs* North, Centre, East and East Polesie (the remaining four populations);

* – p-value < 0.05

** – p-value ≈ 0.05
