# Supplementary material for: Uniparental Genetic Heritage of Belarusians: Encounter of Rare Middle Eastern Matrilineages with a Central European Mitochondrial DNA Pool
Source: PLoS One. 2013 Jun 13;8(6):e66499. doi: 10.1371/journal.pone.0066499 (PMC3681942; doi:10.1371/journal.pone.0066499)
Supplement: Table S5 — Pairwise population Fst calculated from mtDNA haplogroup frequencies in six Belarusian sub-populations. (DOCX) [file pone.0066499.s010.docx]

**Table S5.** Pairwise population Fst calculated from mtDNA haplogroup frequencies in six Belarusian sub-populations.

|  | BeE | BeWP | BeEP | BeW | BeN | BeC |
| --- | --- | --- | --- | --- | --- | --- |
| BeE | 0.00000 |  |  |  |  |  |
| BeWP | 0.00420 | 0.00000 |  |  |  |  |
| BeEP | **0.01417*** | 0.00000 | 0.00000 |  |  |  |
| BeW | 0.00000 | 0.00000 | 0.00000 | 0.00000 |  |  |
| BeN | 0.00484 | 0.00240 | 0.00367 | 0.00000 | 0.00000 |  |
| BeC | **0.01651*** | 0.00890 | 0.00988 | 0.00000 | 0.00683 | 0.00000 |

***** – P-value <0.05;

BeN, BeW, BeC, BeWP, BeEP, BeE – Belarusians from North, West, Central, West Polesie, East Polesie and East sub-regions, respectively
