# Supplementary material for: Uniparental Genetic Heritage of Belarusians: Encounter of Rare Middle Eastern Matrilineages with a Central European Mitochondrial DNA Pool
Source: PLoS One. 2013 Jun 13;8(6):e66499. doi: 10.1371/journal.pone.0066499 (PMC3681942; doi:10.1371/journal.pone.0066499)
Supplement: Table S8 — Pairwise population Fst calculated from NRY haplogroup frequencies in six Belarusian sub-populations. (DOCX) [file pone.0066499.s013.docx]

**Table S8.** Pairwise population Fst calculated from NRY haplogroup frequencies in six Belarusian sub-populations.

|  | BeE | BeWP | BeEP | BeW | BeN | BeC |
| --- | --- | --- | --- | --- | --- | --- |
| BeE | 0.00000 |  |  |  |  |  |
| BeWP | **0.01586*** | 0.00000 |  |  |  |  |
| BeEP | **0.06776*** | **0.02156*** | 0.00000 |  |  |  |
| BeW | 0.00967 | **0.03041*** | **0.05573*** | 0.00000 |  |  |
| BeN | 0.00000 | **0.01844*** | **0.06491*** | 0.00223 | 0.00000 |  |
| BeC | **0.01547*** | 0.00113 | 0.01115 | 0.00922 | **0.01595*** | 0.00000 |

***** – P-value <0.05;

BeN, BeW, BeC, BeWP, BeEP, BeE – Belarusians from North, West, Central, West Polesie, East Polesie and East sub-regions, respectively
